# CT Versus Ultrasound in Young Women With Right Iliac Fossa Pain: A Single‐Centre Retrospective Study

**DOI:** 10.1111/ans.70854

**Published:** 2026-07-12

**Authors:** Anna Pathak, Xinyue Jiang, Jedrzej J. Jaworski, Mohamed Shams, Phill Pearce

**Affiliations:** ^1^ John Radcliffe Hospital Oxford University Hospitals NHS Foundation Trust Oxford UK; ^2^ Buckinghamshire NHS Trust Plastics Surgery Department Stoke Mandeville Hospital Aylesbury Buckinghamshire UK; ^3^ Medical Sciences Division Office, Level 3, Academic Centre University of Oxford Medical School Oxford UK; ^4^ Division of Protein & Nucleic Acid Chemistry MRC Laboratory of Molecular Biology Cambridge UK

**Keywords:** computed tomography, CRP, fever, right iliac fossa pain, ultrasound, WCC, young women

## Abstract

**Background:**

Acute right iliac fossa (RIF) pain is a common presentation in premenopausal women. Compared with men, women are less likely to undergo abdominopelvic computed tomography (CT) and are twice as likely to have a negative diagnostic laparoscopy for appendicitis. However, CT exposes patients to significant ionising radiation—2 years of background exposure—raising lifetime cancer risk to one in 3700. Imaging decisions in this cohort are therefore clinically complex. This study investigated whether rates of negative diagnostic laparoscopy differ between women who underwent no imaging, ultrasound (US), or CT.

**Methods:**

We retrospectively reviewed women aged 16–55 years presenting with acute RIF pain over a 2‐year period at a tertiary referral centre who underwent laparoscopic appendicectomy. Data collected included imaging modality and findings, histopathological diagnosis, initial fever, WCC and CRP. The reference standard was histopathologically confirmed appendicitis. Diagnostic performance of CT and US was assessed using positive predictive value (PPV) and sensitivity.

**Results:**

We identified 502 women, of whom 421 underwent imaging. CT only was performed in 238 patients, US only in 177, both modalities in 6, and 81 had no imaging. US scans were more frequently inconclusive than CT (130 vs. 11). Appendicitis was confirmed histologically in 68.1% of unscanned patients, 48.1% following US and 86.6% following CT. CT demonstrated higher diagnostic accuracy (PPV 92.5%, sensitivity 96.4%) than US (PPV 75.0%, sensitivity 35.5%). Negative appendicectomy rates were significantly higher following US. Raised CRP and WCC correlated with positive histopathology and CT results but less consistently with positive US. Fever was rare (11) and did not correlate with any of the groups.

**Conclusion:**

In young women, the risk of negative laparoscopy must be balanced against radiation exposure from CT. CT or clinical assessment alone may reduce unnecessary appendicectomies compared with US.

## Graphical abstract

In young women with right iliac fossa pain, CTs have a higher positive predictive value for appendicitis than ultrasounds. They also correlate better with raised inflammatory markers. CT could reduce the rate of unnecessary appendicectomies in young women in those without clear clinical signs and symptoms.

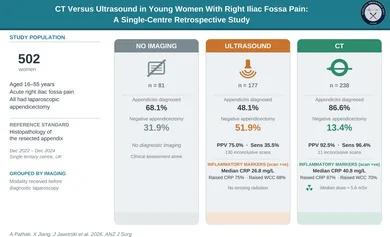

## 1 Introduction

Right iliac fossa (RIF) pain is among the most frequent reasons for presentation to acute general surgery, often raising suspicion for acute appendicitis. Despite advances in diagnostic tools and clinical scoring systems, timely and accurate diagnosis of appendicitis remains a challenge—particularly in women of reproductive age, who are statistically less likely to receive sensitive imaging (abdomino‐pelvic computed tomography (CT) with intravenous contrast) due to the risk of radiation for future pregnancies and are twice as likely to undergo negative diagnostic laparoscopies [1].

Current guidelines on improving diagnostic accuracy—including the 2020 update of the World Society of Emergency Surgery (WSES) Jerusalem guidelines—recommend the use of validated clinical scoring tools such as the Appendicitis Inflammatory Response (AIR) score or the Adult Appendicitis Score (AAS) [2, 3]. These tools stratify patients into high, intermediate, or low risk for appendicitis, guiding decisions around imaging and surgical intervention. For example, high‐risk patients under 40 years of age with classical clinical signs may proceed directly to surgery without imaging, while intermediate‐risk patients may benefit from further imaging and observation. Low‐risk patients, on the other hand, may be safely discharged without imaging, provided appropriate safety‐netting measures are in place [3].

When imaging is indicated for RIF pain, abdomino‐pelvic ultrasonography (US) and abdomino‐pelvic CT with intravenous contrast are the main modalities considered. Other modalities such as abdomino‐pelvic MRI, abdomino‐pelvic non‐contrast CT and low‐dose CT are available but limited due to cost, availability and sensitivity [4]. US offers the advantage of a lack of ionising radiation but has a relatively low sensitivity in detecting appendicitis (36%) [5]. In contrast, CT has been shown in mixed sex adult studies to offer higher sensitivity (95%) and specificity (94%) but carries a higher radiation burden [1]. A typical abdomino‐pelvic CT at Oxford University Hospitals (OUH) NHS Trust delivers a median dose of 5.6 mSv, significantly more than a chest X‐ray (0.02 mSv) or even a transatlantic flight (0.01 mSv) [6]. While the International Commission on Radiological Protection (ICRP) considers the additional cancer risk from exposure low, the risk is higher for patients with larger abdominal circumference and younger patients [7]. The risk to future pregnancies must also be considered in women of reproductive age [8].

Given these trade‐offs between diagnostic yield and radiation risk—particularly among women—and the risks of negative laparoscopy rates, the choice of imaging modality remains contentious.

This study aims to:

1. Compare the number of adult women presenting to a general surgery tertiary referral centre with acute RIF pain who received US, CT, or no imaging prior to diagnostic laparoscopy.
2. Use histological analysis of the appendix removed during surgery to establish the rate of negative diagnostic laparoscopies between patients who received US, CT, or no imaging prior to surgery.
3. Investigate the relationship between CRP, WCC and fever with scan and histopathology outcomes.

We hope to contribute to the debate on the use of cross‐sectional imaging (CT) in patients considered for diagnostic laparoscopy and inform more effective and equitable diagnostic pathways for patients presenting with RIF pain.

## 2 Methods

### 2.1 Study Design and Setting

A retrospective observational study was conducted at the Surgical Emergency Unit, John Radcliffe Hospital, Oxford University Hospitals (OUH) trust, England. Retrospective data were collected from all patients who underwent surgery between 1st December 2022 and 31st December 2024 by three clinical researchers.

### 2.2 Study Population Inclusion and Exclusion Criteria

The study included all patients assigned female at birth, aged 16–55 years, who were referred to the surgical emergency unit with RIF pain between 1st December 2022 and 31st December 2024. Only patients who then proceeded to diagnostic laparoscopy for suspected appendicitis were included in the analysis.

### 2.3 Data Collection

Patients were identified through a retrospective search of electronic health records. Data were extracted from the trust's electronic patient records with support from the Data Analytics Operations Team at OUH Trust.

The following variables were collected:

- Date of presentation to the emergency surgical unit
- Date of operation
- Patient age at presentation
- Self‐reported ethnicity
- Imaging modality used (none, US or CT)
- Reported outcome of imaging (appendicitis confirmed, excluded or inconclusive)
- Final histological diagnosis (appendicitis confirmed, excluded or uncertain)
- Presence of fever (temperature of 38°C (100.4°F) or above)
- Initial White Cell Count (WCC) in ×10^9^/L (billion cells per litre)
- Initial C‐Reactive Protein (CRP) in milligrams per litre (mg/L)

Patients were grouped based on the imaging modality received prior to surgery (USS, CT or no imaging).

### 2.4 Outcomes and Statistical Analysis

The primary outcome was whether pre‐operative imaging correctly confirmed appendicitis. Imaging reports labelled “inconclusive” for appendicitis and not providing an alternative diagnosis were interpreted as no evidence of appendicitis. The secondary outcome was the rate of negative diagnostic laparoscopy, defined as cases where the excised appendix did not show histological evidence of appendicitis.

The tertiary outcomes were presence of fever, abnormal WCC and CRP.

Data were analysed descriptively to visualise distributions of nominal, mutually exclusive categories. Group comparisons (e.g., negative laparoscopy rates by imaging modality) were carried out using Chi‐squared tests [9].

### 2.5 Ethics and Dissemination

The study was registered locally as a service evaluation. No direct patient contact occurred, and all data were anonymised prior to analysis. Data were stored on a password‐protected spreadsheet.

## 3 Results

A total of 502 patients met the inclusion criteria for this study with no exclusions. The mean age was 31.9 years (±11.5) with a range of 16–55 years (Table 1). The oldest group were CT (41) and the youngest were US only (22).

**TABLE 1:** Baseline patient demographic characteristics (*n* = 502).

	No imaging (*n* = 81)	US only (*n* = 177)	CT only (*n* = 238)	Both modalities (*n* = 6)	Total (*n* = 502)
Characteristics	No. (%)	No. (%)	No. (%)	No. (%)	No. (%)
**Age, years (median, range)**	24.0 (16–42)	22.0 (16–49)	41.0 (19–55)	28.0 (19–35)	31.0 (16–55)
**Ethnicity**					
Any white background	53 (65.4%)	119 (67.2%)	160 (67.2%)	5 (83.3%)	337 (67.1%)
Asian	3 (3.7%)	13 (7.3%)	15 (6.3%)	0 (0.0%)	31 (6.2%)
Black	2 (2.5%)	1 (0.6%)	7 (2.9%)	0 (0.0%)	10 (2.0%)
Mixed	1 (1.2%)	5 (2.8%)	3 (1.3%)	0 (0.0%)	9 (1.8%)
Other	0 (0.0%)	2 (1.1%)	4 (1.7%)	0 (0.0%)	6 (1.2%)
Not stated	22 (27.2%)	37 (20.9%)	49 (20.6%)	1 (16.7%)	109 (21.7%)

Abbreviations: CT, computed tomography; US, ultrasound.

The cohort was stratified into four mutually exclusive groups based on type and presence of preoperative imaging. Of the 502 patients, 421 (83.9%) had preoperative imaging, of which 238 (47.4%) had CT only and 177 (35.2%) had US only; 6 (1.2%) patients had both. The remaining 81 (16.1%) of patients received no preoperative imaging (Table 1).

Overall, histopathology confirmed appendicitis in 357 (71.1%) of cases (Table 2). Negative histopathology in 139 patients confirmed a negative appendicectomy rate of 27.7% in our sample, while in five (1.0%) samples, histopathology was uncertain. In one remaining case, the histopathology report could not be found.

**TABLE 2:** Histopathology by imaging result.

	US positive (*n* = 45)	US inconclusive (*n* = 130)	CT positive (*n* = 212)	CT inconclusive (*n* = 11)	CT negative (*n* = 15)	Total (*n* = 502)
	No. (%)	No. (%)	No. (%)	No. (%)	No. (%)	No. (%)
Histopathology diagnosis						
Positive (+)	34 (76%)	52 (40%)	197 (93%)	3 (27%)	7 (47%)	357 (71.1%)
Negative (−)	11 (24%)	78 (60%)	11 (5%)	7 (64%)	8 (53%)	139 (27.7%)
Uncertain (0)	0 (0%)	0 (0%)	3 (1%)	1 (9%)	0 (0%)	5 (1%)
N/A	0 (0%)	0 (0%)	1 (0%)	0 (0%)	0 (0%)	1 (0.2%)

### 3.1 Imaging Results (Figure 1)

**FIGURE 1:**
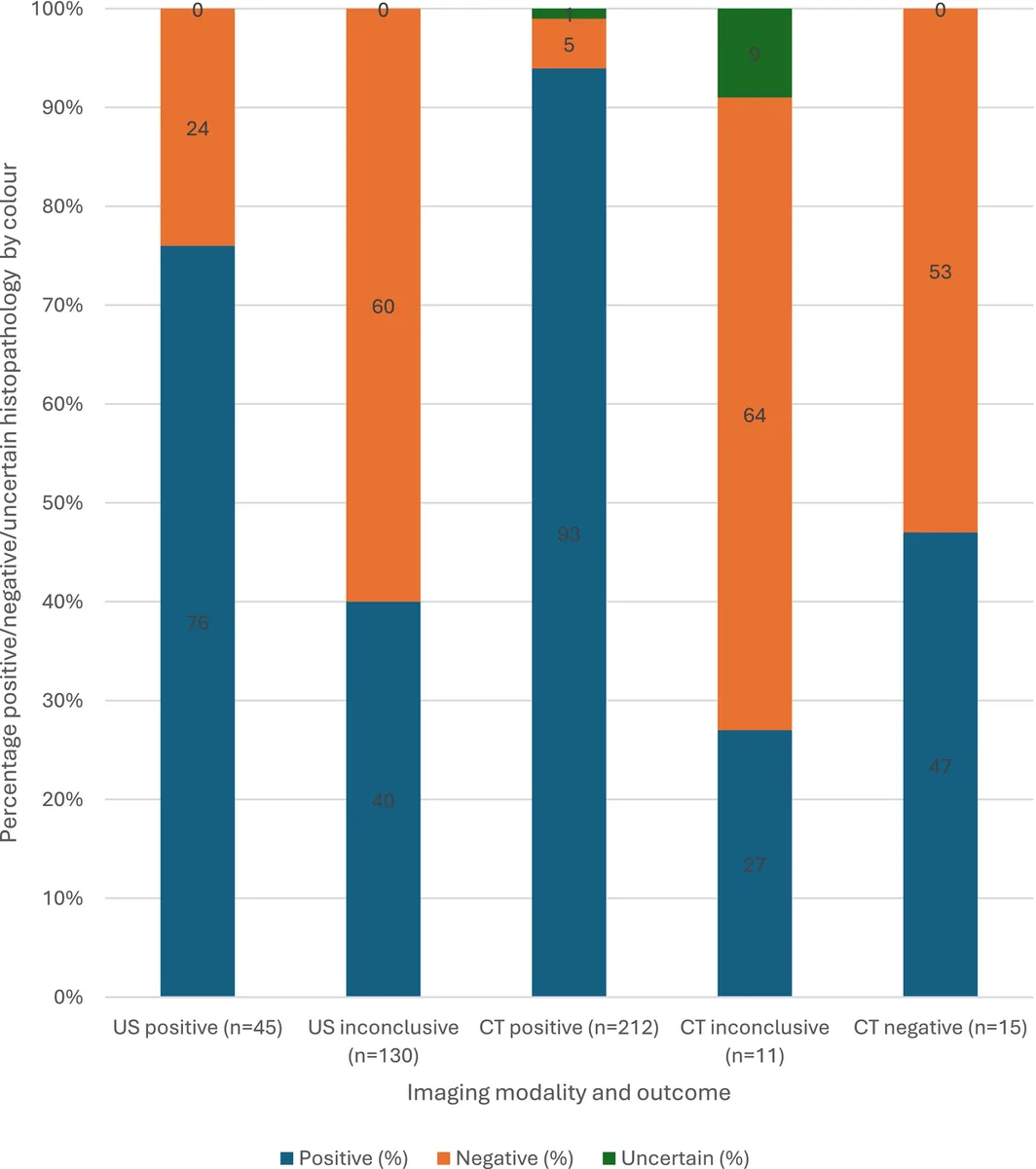
Histopathological results by preoperative imaging group, showing the proportion of confirmed appendicitis, negative appendicectomies and inconclusive results.

The results of pre‐operative imaging were analysed and categorised as Positive for appendicitis, Negative for appendicitis, or Inconclusive. In patients who had CT preoperatively, 90.1% of scans were positive for appendicitis, compared to 22.8% of US scans. US scans also had a higher rate of uncertainty, as 67.7% of US scans were inconclusive compared to 5.9% of CT scans.

### 3.2 Negative Appendicectomy Rate

The primary outcome, the negative appendicectomy rate (NAR), was calculated using histopathology reports and was analysed by imaging modality. Uncertain histopathology was excluded from the calculation of NAR. Histopathology results by imaging modality are represented in Figure 1. The US‐only group had the highest rate of negative appendicectomy at 51.9%, followed by no imaging (NI) at 30.7%. Patients who had CTs preoperatively had the lowest NAR of 11.5%. Statistical comparison of the NAR between imaging modalities using a chi‐squared test revealed a significant difference between groups (*χ* ^2^ = 77.8635, *p* < 0.00001). This was further analysed by pairwise post hoc testing with Bonferroni correction, establishing a significance level of *p* = 0.0167. Significant differences were found in NAR in all comparisons; US and CT (*p* < 0.00001), US and NI (*p* < 0.0001) and CT and NI (*p* < 0.01).

### 3.3 Diagnostic Performance of Imaging Modalities (Table 3)

**TABLE 3:** Clinical characteristics by imaging result.

Clinical characteristics	US positive (*n* = 45)	US inconclusive (*n* = 130)	CT positive (*n* = 212)	CT inconclusive (*n* = 11)	CT negative (*n* = 15)
	No. (%)	No. (%)	No. (%)	No. (%)	No. (%)
**Histopathology diagnosis**					
Positive (+)	34 (76%)	52 (40%)	197 (93%)	3 (27%)	7 (47%)
Negative (−)	11 (24%)	78 (60%)	11 (5%)	7 (64%)	8 (53%)
Uncertain (0)	0 (0%)	0 (0%)	3 (1%)	1 (9%)	0 (0%)
N/A	0 (0%)	0 (0%)	1 (0%)	0 (0%)	0 (0%)
**Inflammatory markers**					
1st CRP (median, range)	26.8 (0.2–226.2)	11.5 (0.2–299.1)	40.8 (0.2–472)	47.6 (2.9–553.8)	2.2 (0.4–109.6)
CRP normal (< 5)	11 (25%)	39 (32%)	26 (13%)	2 (18%)	8 (57%)
CRP increased (≥ 5)	33 (75%)	82 (68%)	180 (87%)	9 (82%)	6 (43%)
1st white cell count (median, range)	12.9 (4.8–20.9)	11 (3.8–25.3)	13.5 (2–27.9)	12.4 (7.8–18.1)	9.5 (4.8–23.1)
WCC normal (< 11)	14 (32%)	61 (50%)	60 (30%)	3 (27%)	9 (64%)
WCC increased (≥ 11)	30 (68%)	60 (50%)	141 (70%)	8 (73%)	5 (36%)
**Clinical observations—fever**					
Yes (+)	1 (2%)	5 (4%)	5 (2%)	0 (0%)	0 (0%)
No (−)	44 (98%)	125 (96%)	207 (98%)	11 (100%)	15 (100%)

Abbreviations: CRP, C‐reactive protein; CT, computed tomography; US, ultrasound.

Diagnostic performance of US and CT was investigated by calculating positive predictive value (PPV) and sensitivity. “Inconclusive” imaging findings were included in the false negative rate in the calculation of sensitivity. Uncertain histopathology was excluded from these calculations. CT was found to have a PPV of 92.5% and sensitivity of 96.4%, both of which were higher than US, which had a PPV of 75.0% and a sensitivity of 35.5%. Interpretable histological outcomes by imaging result are summarised in Figure 2.

**FIGURE 2:**
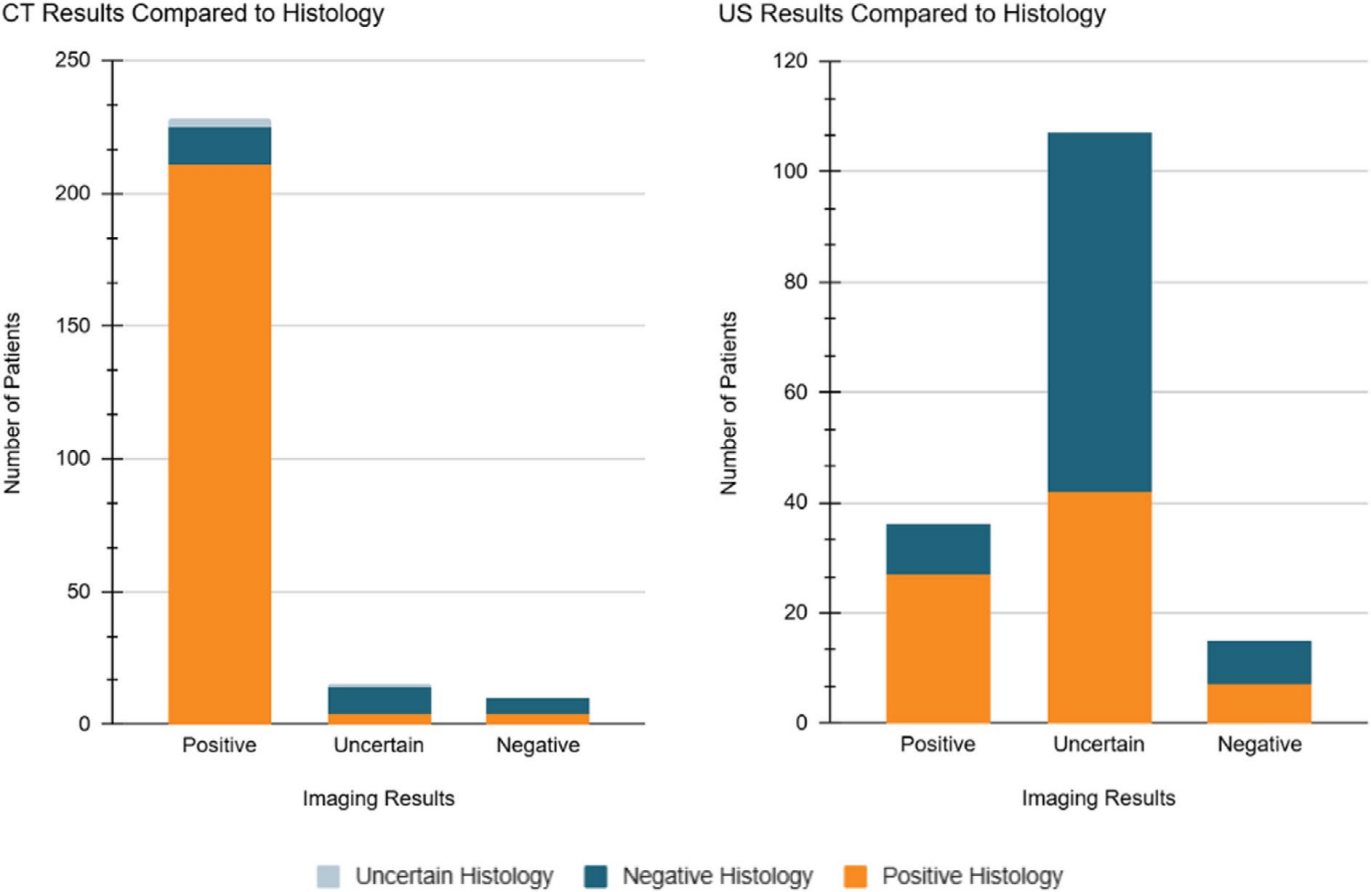
CT and US results (positive, negative or inconclusive for diagnosis of appendicitis on *X*‐axis) displayed with colour‐coded histopathology results (confirmed positive, negative or uncertain evidence of appendicitis on histopathological specimen).

### 3.4 Fever

Across all five groups, fever was present in only 0%–4% of patients. US inconclusive (4%) had a *higher* rate of fever than US positive (2%) or CT positive (2%). CT inconclusive and CT negative both show 0% fever, but these are small groups (*n* = 11 and *n* = 15) (Table 3).

### 3.5 Blood Results

CT positive patients had the highest inflammatory markers of any group: median CRP 40.8 (range 0.2–472), with 87% having an elevated CRP and 70% an elevated WCC (Table 3). This group also had the highest histopathology‐confirmed positive rate (93%) (Table 4).

**TABLE 4:** Clinical characteristics by histopathology diagnosis.

	Histopathology positive (+) (*n* = 357)	Histopathology negative (−) (*n* = 139)	Histopathology uncertain (0) (*n* = 5)
Key parameters	No. (%)	No. (%)	No. (%)
**Inflammatory markers**			
1st CRP (median, range)	33 (0.2–472)	12.3 (0.2–553.8)	77.8 (59.4–275.8)
CRP normal (< 5)	59 (17%)	41 (31%)	0 (0%)
CRP increased (≥ 5)	285 (83%)	90 (69%)	5 (100%)
1st white cell count (median, range)	13.4 (2–27.2)	10.5 (3.8–23.1)	16.4 (7.8–18)
WCC normal (< 11)	98 (29%)	72 (55%)	2 (40%)
WCC increased (≥ 11)	241 (71%)	58 (45%)	3 (60%)
**Fever**			
Yes (+)	7 (2%)	6 (4%)	0 (0%)
No (−)	350 (98%)	133 (96%)	5 (100%)

Abbreviation: CRP, C‐reactive protein.

US positive patients showed a less extreme tendency towards higher inflammatory markers: median CRP 26.8, 75% elevated CRP, 68% elevated WCC and 76% histopathology‐positive (Table 3).

CT negative patients had the lowest inflammatory markers—median CRP just 2.2, with only 43% elevated and only 36% with raised WCC. However, 47% of this group were still histopathology‐positive.

US inconclusive (the largest group, *n* = 130) had CRP elevated in 68% and WCC elevated in 50% (Table 3). Only 40% were histopathology positive (Table 4).

CT inconclusive (*n* = 11) had a high median CRP (47.6, the highest in the table) and 82% with elevated CRP, yet only 27% were histopathology positive (Table 4).

## 4 Discussion

Our study aligns with findings in previous literature indicating the superior diagnostic accuracy for appendicitis of CT (94%–98%) over US (36%) in adult populations [5]. CT is also increasingly affordable, available and safer with progressing technology.

CT reports were more likely to be clearly positive or negative for appendicitis. In contrast, a substantial proportion of US reports in our cohort were indeterminate. This diagnostic limitation of US reduces its utility in guiding surgical decisions, particularly when the differential diagnosis is broad. In our statistical analysis, an inconclusive report was taken to be negative for appendicitis as it did not provide positive evidence. In clinical practice as well, inconclusive reports or those that did not provide an alternate diagnosis don't contribute to the decision to progress to theatre. In addition, the diagnostic yield of US is heavily operator‐dependent and is more challenging in patients with larger abdominal circumference, further complicating its reliability.

Interestingly, patients who did not undergo imaging prior to surgery had a higher rate of appendicitis confirmed with histopathology compared to those who received US. This may reflect clinically diagnostic presentations of appendicitis prompting surgeons to proceed directly to operation without imaging. This supports current WSES guidelines, which allow for immediate surgery in high‐risk patients with classic clinical signs, particularly in those under 40 years old [3]. In these cases, US may not offer significant additional value and could potentially delay definitive treatment.

The presence of both “inconclusive” imaging results introduces complexity into the analysis of diagnostic accuracy. Specifically, categorising “uncertain” or “inconclusive” findings poses a challenge when calculating sensitivity, as these results may not clearly classify as true positives or negatives. Future studies should consider standardised criteria for interpreting equivocal imaging and may benefit from excluding or separately analysing indeterminate cases to more accurately assess diagnostic performance.

Additionally, a subgroup of patients underwent both US and CT, offering an opportunity for further analysis on imaging sequencing and its impact on diagnostic accuracy and clinical decision‐making. For example, evaluating whether US followed by CT alters outcomes or leads to delayed intervention could inform more efficient imaging pathways.

### 4.1 Clinical Symptoms, Signs and Blood Results

Fever showed almost no relationship with any outcome and was rare across every category. This could be because it was not coded for accurately either.

Blood results were much more descriptive. A higher CRP and WCC count were generally consistent with positive scan results for both CT and US. This is clearest for CT (CT positive vs. CT negative is a clean split), but the relationship is noisier for US (US inconclusive has fairly high CRP elevation despite a low confirmed‐positive rate). The CT inconclusive group's blood results don't fit the rest of the pattern at all with a high CRP result—though the sample was small (11 patients). CT negative scans missed nearly half of confirmed cases despite those patients generally having low inflammatory markers—suggesting blood results alone wouldn't have flagged these missed cases either.

For US inconclusive, CRP and WCC were elevated in around half of patients and only 40% were histopathology‐positive, so blood markers here are weak discriminators.

### 4.2 Limitations

This study is limited by its retrospective design and reliance on clinical coding of diagnoses. Unfortunately, clinical symptoms such as pain and nausea were not coded accurately so could not be extracted retrospectively. Patient imaging was not randomised and was based on clinical judgment and symptoms, introducing selection bias. Inter‐operator variability in US operation and interpretation was not controlled for, and the decision to image or proceed directly to surgery was likely influenced by individual clinician judgment, expertise and local protocols, introducing potential selection bias. Further studies could stratify patients based on their clinical symptoms.

Specificity could not be calculated due to the unknown number of true negative appendicitis presentations excluded from our study population (everyone who did not progress to surgery was excluded). A future study should be prospective and follow everyone having a scan for appendicitis, whether they progress to surgery or not.

We did not collect data on intra‐operative findings as this was considered less sensitive for a diagnosis of appendicitis than histopathology so does not advance our study aims.

## 5 Conclusion

CT offers significantly superior diagnostic performance compared to US in women presenting with RIF pain, with higher sensitivity and PPV, and is associated with a lower rate of negative diagnostic laparoscopies. While CT carries a modest radiation risk—particularly relevant in women of reproductive age and in younger and thinner patients—this risk must be balanced against the potential morbidity of unnecessary surgery, especially given the risks of diagnostic laparoscopy.

US, despite its safety profile, remains limited in its ability to positively predict appendicitis, and is frequently inconclusive. As such, US should be reserved for selected cases where alternative diagnoses (e.g., gynaecological pathology) are being considered. For most intermediate‐risk presentations, cross‐sectional imaging with CT should be prioritised.

Clinical assessment and validated scoring systems such as the Adult Appendicitis Score (AAS) remain essential to guide imaging decisions [2]. In patients with a clear clinical picture of appendicitis, imaging may be unnecessary and could delay definitive management. Given the relatively low radiation dose from CT at our institution, improved patient counselling could help inform shared decision‐making, particularly for women in whom diagnostic uncertainty often leads to more conservative imaging choices.

Further research should explore outcomes in patients who did not undergo surgery and investigate whether imaging modality choice impacts the management of complicated versus uncomplicated appendicitis. Low‐dose CT and MRI may become more viable alternatives with improvements in technology and reduction in cost. Prospective studies are also needed to evaluate the diagnostic and operational value of sequential imaging strategies and refine clinical pathways for this common surgical presentation.

## Funding

The authors have nothing to report.

## Ethics Statement

All data were anonymised, and ethics approval was not needed.

## Conflicts of Interest

The authors declare no conflicts of interest.

## Data Availability

The data that support the findings of this study are available on request from the corresponding author. The data are not publicly available due to privacy or ethical restrictions.
